# Retraction Note: Mass–Energy Equivalence Extension onto a Superfluid Quantum Vacuum

**DOI:** 10.1038/s41598-020-80949-z

**Published:** 2021-01-06

**Authors:** Amrit Srečko Šorli

**Affiliations:** Bijective Physics Institute, Idrija, Slovenia

Retraction of: *Scientific Reports* 10.1038/s41598-019-48018-2, published online 13 August 2019

The Editors have retracted this Article.

After publication, concerns were raised about this Article, in particular with respect to the theoretical foundation of and the rationale for the model introduced in the paper, the lack of literature support for many of the claims made, the inappropriate use of the supportive literature, the inadequate level of methodological detail provided to allow for replication of the analyses presented, and the lack of evidence in support of the described model. Post-publication peer review has confirmed these concerns. The Editors no longer have confidence in the results and conclusions of this Article.

Amrit Srečko Šorli disagrees with the retraction.

